# Comprehensive performance evaluation of four chemiluminescence immunoassays for detecting vitamin D deficiency across diverse clinical conditions

**DOI:** 10.1371/journal.pone.0329796

**Published:** 2025-08-04

**Authors:** Tasneem AlHamad, Salma Younes, Dayana El Chaar, Parveen B. Nizamuddin, Eiman Al Mohannadi, Asmaa Alghanim, Nadin Younes, Nader Al-dewik, Wanida Laiwattanapaisal, Palanee Ammaranond, Phillip Hawken, Laith J. Abu-Raddad, Gheyath K. Nasrallah

**Affiliations:** 1 Biomedical Sciences Department, College of Health Sciences, Qatar University, Doha, Qatar; 2 Biomedical Research Center, Qatar University, Doha, Qatar; 3 Department of Research, Women’s Wellness and Research Center, Hamad Medical Corporation, Doha, Qatar; 4 Centre of Excellence for Biosensors and Bioengineering (CEBB), Department of Clinical Chemistry, Faculty of Allied Health Sciences, Chulalongkorn University, Bangkok, Thailand; 5 Department of Clinical Chemistry, Faculty of Allied Health Sciences, Chulalongkorn University, Bangkok, Thailand; 6 Department of Transfusion Medicine and Clinical Microbiology, Faculty of Allied Health Sciences, Chulalongkorn University, Bangkok, Thailand; 7 Department of Immunology, Sidra Medicine, Doha, Qatar; 8 Infectious Disease Epidemiology Group, Weill Cornell Medicine–Qatar, Cornell University, Qatar Foundation Education City, Doha, Qatar; 9 World Health Organization Collaborating Centre for Disease Epidemiology Analytics on HIV/AIDS, Sexually Transmitted Infections, and Viral Hepatitis, Weill Cornell Medicine–Qatar, Cornell University, Qatar Foundation, Education City, Doha, Qatar; 10 Department of Healthcare Policy and Research, Weill Cornell Medicine, Cornell University, New York, New York, United States of America; University of Toronto Department of Laboratory Medicine and Pathobiology, CANADA

## Abstract

**Background/Objectives:**

Vitamin D deficiency is a significant global health concern, requiring accurate diagnosis. This study evaluates the performance of four chemiluminescent immunoassay (CLIA) platforms; Snibe, Roche, DiaSorin, and Architect for vitamin D measurement.

**Methods:**

A total of 345 serum samples, selected to represent a broad range of vitamin D levels and diverse health conditions, including pregnancy, chronic kidney disease, and osteoporosis, were analyzed using four platforms. Diagnostic metrics, including sensitivity, specificity, and overall percent agreement (OPA), were calculated. Spearman’s rank correlation and bias assessment were performed to evaluate inter-assay agreement.

**Results:**

Spearman’s rank correlations were strong to very strong across the platforms, ranging from r = 0.924 to r = 0.969, reflecting high inter-assay concordance. Pairwise comparisons indicated that Snibe demonstrated high specificity (98–99%) and strong agreement with DiaSorin (κ = 0.91), while DiaSorin maintained a favorable balance between sensitivity (86–98%) and specificity (94–99%). Roche showed consistent diagnostic characteristics, with sensitivity ranging from 93–99% and specificity from 85–96%. Architect exhibited high sensitivity (97–99%) but relatively lower specificity (81–92%).

**Conclusions:**

All platforms demonstrated robust diagnostic performance. Snibe showed notably high specificity, while DiaSorin offered balanced sensitivity and specificity. These findings underscore the relative strengths of each platform and support their use in clinical evaluation of vitamin D status.

## 1. Introduction

Vitamin D plays a critical role in maintaining bone health, immune function, and metabolic regulation [1–3]. Despite its significance, vitamin D deficiency is a global health concern, affecting individuals across all age groups and geographic regions [4]. Insufficient levels of serum 25-hydroxyvitamin D [25(OH)D], the primary marker for assessing vitamin D status, are associated with skeletal disorders such as rickets and osteomalacia, as well as increased risks of autoimmune diseases, cardiovascular conditions, and metabolic syndromes [1–3]. Early and accurate diagnosis of vitamin D deficiency is essential for effective treatment and prevention.

The measurement of 25(OH)D levels is commonly performed using various analytical techniques, including chemiluminescent immunoassays (CLIAs), enzyme-linked immunosorbent assays (ELISA), and liquid chromatography-tandem mass spectrometry (LC-MS/MS) [5]. Among these, LC-MS/MS is considered the gold standard due to its high specificity and ability to differentiate between vitamin D metabolites. However, the complexity, cost, and lack of widespread availability of LC-MS/MS have limited its use in routine clinical practice [6–8].

CLIAs have gained popularity due to their automation, cost-effectiveness, and rapid processing times. Platforms such as Roche Cobas, Diasorin LIAISON®, Snibe MAGLUMI, and Abbott Architect are commonly used, yet significant inter-assay variability and a lack of standardization across these systems compromise result reliability and clinical interpretation [9]. Most existing evaluations focus on individual platform performance, with limited comparative data on the concordance and variability among these widely used assays.

To address this gap, this study evaluates and compares the performance of four leading CLIA platforms, Roche, Diasorin, Snibe, and Architect, in measuring serum 25(OH)D levels. By analyzing sensitivity, specificity, and inter-assay agreement, this research highlights discrepancies and provides practical insights for optimizing diagnostic strategies. This study offers a unique contribution by evaluating four leading chemiluminescent immunoassays (CLIAs) for serum 25-hydroxyvitamin D [25(OH)D] measurement, including the Snibe platform, which has not been comprehensively assessed in the literature. Additionally, the inclusion of a diverse range of clinical samples, spanning various vitamin D levels and clinical conditions such as pregnancy, chronic kidney disease, and osteoporosis, ensures a robust and clinically relevant analysis. These features position our study as a significant advancement in the field of vitamin D diagnostics, providing valuable insights for both clinical and laboratory applications.

## 2. Methods

### 2.1. Sample collection and ethical approval

A total of 345 serum samples were obtained from the Qatar Biobank for this study. Qatar Biobank is a national health initiative that collects biological samples and associated health data from participants to support population-level research. Written informed consent was obtained from all participants prior to their enrollment in the biobank. No minors were involved in this study. All samples were collected and stored in a highly regulated environment to ensure sample quality and integrity. For this study, the samples were provided in aliquots and stored at −80°C to prevent degradation.

The samples and associated data were accessed for research purposes following ethical approval granted by the Institutional Review Board at Qatar University (IRB# QU-IRB 2011-E/23) on 14 January 2024. The authors received fully de-identified data, including only dummy sample IDs and limited, non-identifying demographic details. No personally identifiable information (PII) was accessible during or after data collection, ensuring strict adherence to confidentiality and ethical research standards.

The study cohort was carefully selected to encompass a diverse range of vitamin D levels, including states of deficiency, insufficiency, sufficiency, and toxicity. The dataset also included individuals with conditions significantly influenced by vitamin D levels, such as pregnancy, chronic kidney disease, and osteoporosis.

### 2.2. Diagnostic assay methodologies

The vitamin D concentrations in serum samples were measured using three advanced chemiluminescence immunoassay (CLIA) platforms: Roche Cobas e6000, DiaSorin LIAISON®, and Snibe Maglumi 25-OH Vitamin D assay. Each platform operates on distinct technological principles that ensure precision and reliability.

#### 2.2.1. *Roche Cobas e6000 analyzer.*

The Roche Cobas e6000 analyzer utilizes a competitive immunoassay technique [10], wherein serum 25(OH)D binds with biotin-labeled vitamin D and ruthenium-labeled vitamin D binding protein. The complex is captured on streptavidin-coated microparticles, and a chemiluminescent reaction is measured using a photomultiplier. Results are calculated using an instrument-specific calibration curve derived from a master curve provided by reagent barcodes [11,12]. This assay is widely regarded for its robustness and accuracy in quantifying serum vitamin D levels across a clinically relevant range.

#### 2.2.2. *DiaSorin LIAISON*^®^ *assay.*

The DiaSorin LIAISON® CLIA operates on a direct competitive assay principle. During the initial incubation, 25(OH)D is released from its binding protein and binds to specific antibodies immobilized on the solid phase. Following incubation with a chemiluminescent tracer, the emitted light is measured in relative light units (RLUs), which inversely correlate with vitamin D concentrations. Vitamin D levels are categorized as deficiency (<20 ng/mL), insufficiency (20–30 ng/mL), sufficiency (>30 ng/mL), and toxicity (>100 ng/mL), facilitating comprehensive clinical interpretation.

#### 2.2.3. *Snibe Maglumi 25-OH Vitamin D assay.*

The Snibe Maglumi 25-OH Vitamin D assay measures serum 25(OH)D using a sandwich assay launched for small molecule compounds. Serum samples are incubated with a displacing reagent, FITC-labeled monoclonal antibodies, and magnetic microbeads coated with anti-FITC antibodies [13]. After several wash cycles, a chemiluminescent reaction is triggered, and the emitted signal is quantified using a photomultiplier. The assay ensures precision through rigorous internal quality control and external validation, aligning with the same clinical cut-off ranges as the DiaSorin assay.

### 2.3. Statistical analysis

All statistical analyses were performed using GraphPad Prism version 9.3.1 (San Diego, CA, USA). The Shapiro-Wilk test was employed to assess the normality of the dataset, and nonparametric tests, including the Kruskal-Wallis test, were applied due to the absence of normal distribution. Data for numerical values were presented as medians and interquartile ranges (IQR), with a p-value <0.05 considered statistically significant. Diagnostic performance metrics, including sensitivity, specificity, positive predictive value (PPV), negative predictive value (NPV), and overall percent agreement (OPA), were calculated and reported with 95% confidence intervals (CIs). These parameters were determined using a cut-off of 30 ng/mL, where serum 25(OH)D levels <30 ng/mL were classified as positive for vitamin D deficiency and levels ≥30 ng/mL were classified as negative. Cohen’s Kappa coefficient was used to assess the level of agreement between assays, with values interpreted as follows: < 0.40 indicated poor agreement, 0.40–0.59 indicated fair agreement, 0.60–0.74 indicated good agreement, and ≥0.75 indicated excellent agreement. Concordance analysis between assays was further evaluated with Bias Assessment. Correlation between assays was assessed using Spearman’s rank correlation coefficient, with values categorized as very weak (0.00–0.19), weak (0.20–0.39), moderate (0.40–0.59), strong (0.60–0.79), and very strong (0.80–1.00).

## 3. Results

### 3.1. Vitamin D level distribution

**Fig 1:**
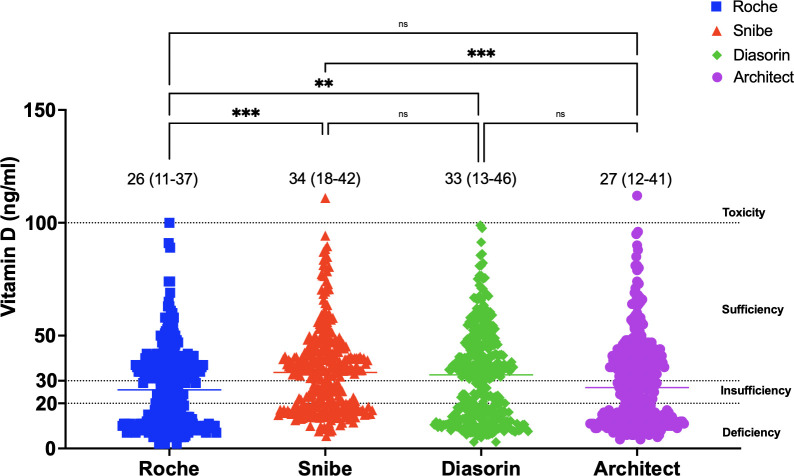
Distribution of vitamin D levels across four chemiluminescent immunoassay platforms (Roche, Snibe, DiaSorin, and Architect). Median values with interquartile ranges (IQR) are indicated for each platform: Roche (26 ng/mL, IQR: 11–37), Snibe (34 ng/mL, IQR: 18–42), DiaSorin (33 ng/mL, IQR: 13–46), and Architect (27 ng/mL, IQR: 12–41). Significant differences are denoted as follows: ***p < 0.001 (very significant), **p = 0.001 to 0.01 (very significant), *p = 0.01 to 0.05 (significant), and ns (p ≥ 0.05; not significant). The horizontal dotted lines represent clinical thresholds for vitamin D classification: deficiency (<20 ng/mL), insufficiency (20–30 ng/mL), sufficiency (>30 ng/mL), and toxicity (>100 ng/mL).

Median values and interquartile ranges (IQR) demonstrated variability among the assays (Fig 1). The Snibe assay reported the highest median value of 33 ng/mL (IQR: 18–42), followed by DiaSorin with a median of 32 ng/mL (IQR: 13–46). Roche recorded a lower median of 26 ng/mL (IQR: 11–37), while Architect presented a median of 27 ng/mL (IQR: 12–41). Statistical analysis revealed significant differences between some assays. Snibe exhibited significantly higher values compared to Roche (p < 0.001). However, no significant differences (ns) were observed between DiaSorin and Snibe or between DiaSorin and Architect, suggesting comparable results among these methods. Detailed data on the vitamin D level distribution across all assays are provided in S1 Table.

### 3.2. Correlation analysis

**Fig 2:**
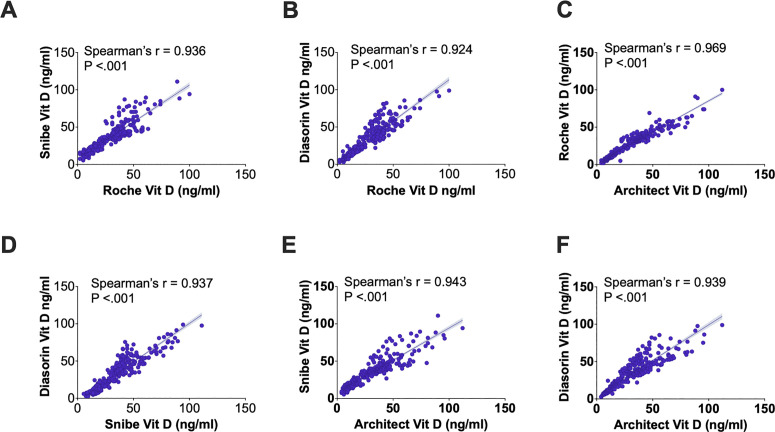
The figure illustrates the Spearman’s correlation between vitamin D measurements across six chemiluminescent immunoassay platform pairings. The scatterplots, accompanied by linear regression lines, demonstrate significant correlations for all comparisons (P < 0.001), with Spearman’s rank correlation coefficients (r) ranging from strong to very strong. Snibe and Roche (A) achieved a correlation of r = 0.936, while DiaSorin and Roche (B) showed a slightly lower, though still strong, correlation of r = 0.924. The highest agreement was observed between Roche and Architect (C), with a correlation of r = 0.969, underscoring exceptional consistency. DiaSorin and Snibe (D) demonstrated a correlation of r = 0.937, while Snibe and Architect (E) achieved a slightly higher correlation of r = 0.943. DiaSorin and Architect (F) also displayed robust concordance, with a correlation of r = 0.939.

Spearman’s rank correlation analysis (Fig 2) demonstrated very strong correlations among all assay comparisons, with correlation coefficients (r) ranging from 0.924 to 0.969 (P < 0.001), reflecting significant concordance in vitamin D measurements.

### 3.3. Bias assessment

**Fig 3:**
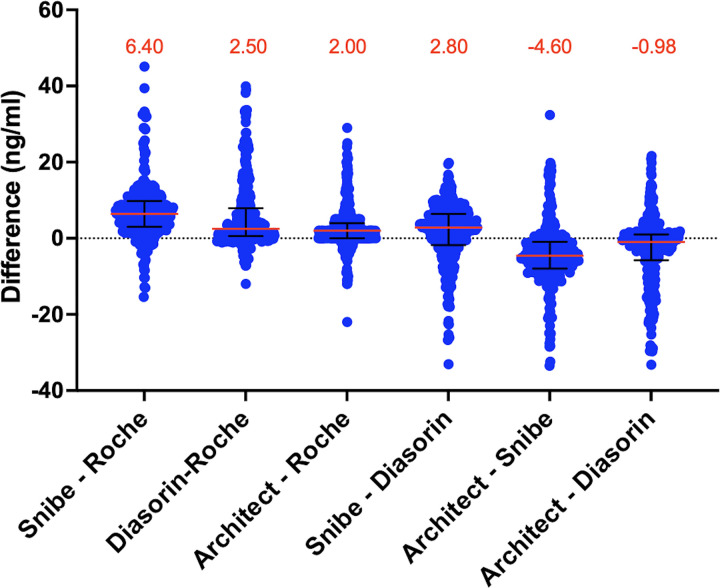
Bias assessment comparing vitamin D measurements across chemiluminescent immunoassay platforms. Median differences (shown in red text and represented by the red horizontal bars) and interquartile ranges (IQR, shown as black vertical bars) are presented for six pairwise comparisons between assay platforms. Differences represent the values obtained by subtracting one assay from another for each sample. Positive median differences indicate higher readings from the first assay in the pair, while negative values indicate lower readings. This plot illustrates the degree of bias and variability between each assay pair, highlighting the closest agreement observed between Architect and DiaSorin.

Bias assessment (Fig 3) presents the median differences along with interquartile ranges (IQR) between the assays, reflecting varying levels of agreement. The closest agreement was observed between Architect and DiaSorin, with a median bias of –0.98 ng/mL (IQR: –5.75 to 1.00), indicating a slight tendency for Architect to measure lower values. In contrast, a larger positive bias was noted between Snibe and Roche, with a median difference of 6.40 ng/mL (IQR: 3.02 to 9.80), suggesting that Snibe generally reports higher values. Architect also showed consistent negative biases compared to Snibe (median: –4.60 ng/mL, IQR: –7.95 to –0.95), reinforcing its overall conservative measurement pattern relative to these assays. These findings align well with the median bias distributions depicted in Fig 3 and highlight systematic differences among the platforms.

### 3.4. Diagnostic performance metrics

The diagnostic performance of Roche, Snibe, DiaSorin, and Architect assays was assessed against various reference methods. Overall, Architect consistently demonstrated strong sensitivity across comparisons, while Snibe excelled in specificity and balanced overall performance. DiaSorin also showed high diagnostic performance, particularly when compared against Snibe.

Using Roche as the reference, Architect exhibited the highest sensitivity (97%) and a specificity of 92%, resulting in an OPA of 94% and Cohen’s Kappa of 0.89, indicating excellent concordance. DiaSorin followed with a sensitivity of 89% and specificity of 98%, achieving an OPA of 93% and a Kappa of 0.86. Snibe demonstrated the lowest sensitivity (84%) in this comparison, although it maintained excellent specificity (99%), yielding an OPA of 91% and Kappa of 0.82. These findings highlight Architect as the strongest performer against Roche, with DiaSorin offering a balanced sensitivity and specificity. Snibe, while highly specific, displayed the lowest sensitivity, making it less consistent in detecting true positives.

Using Architect as the reference, Roche demonstrated the highest sensitivity (93%) with a strong specificity of 96%, resulting in an OPA of 94% and Cohen’s Kappa of 0.89, indicating excellent concordance. DiaSorin followed with slightly lower sensitivity (86%) but maintained excellent specificity (99%), yielding an OPA of 92% and Kappa of 0.83. Snibe exhibited the lowest sensitivity (81%) in this comparison, despite its excellent specificity (99%), resulting in an OPA of 89% and Kappa of 0.78. These findings emphasize that Roche provides the most balanced performance against Architect, while DiaSorin maintains excellent specificity with slightly reduced sensitivity. Snibe, though highly specific, shows greater variability in sensitivity.

Using Snibe as the reference, both Architect and Roche exhibited comparable sensitivity, each achieving 99%. However, Roche showed a slightly higher specificity (85%) compared to Architect (81%). This resulted in an OPA of 91% and Cohen’s Kappa of 0.82 for Roche, while Architect achieved a slightly lower OPA of 89% and Kappa of 0.78. DiaSorin demonstrated a well-balanced performance with a sensitivity of 98% and the highest specificity (94%), leading to the highest OPA of 96% and Kappa of 0.91, making it the best overall performer in this comparison.

Using DiaSorin as the reference, Architect demonstrated the highest sensitivity (99%) but had a lower specificity (85%), resulting in an OPA of 92% and Cohen’s Kappa of 0.83. Roche showed excellent sensitivity (98%) and slightly higher specificity (89%) compared to Architect, yielding an OPA of 93% and Kappa of 0.86. Snibe exhibited the most balanced performance with a sensitivity of 93% and the highest specificity (98%), achieving the highest OPA of 96% and Cohen’s Kappa of 0.91.

## 4. Discussion

Accurate assessment of vitamin D status is essential for diagnosing deficiency and ensuring appropriate clinical interventions, particularly in at-risk populations such as pregnant women, patients with chronic kidney disease, and individuals with osteoporosis [1,2,4,14,15]. While CLIAs are widely used, variability among diagnostic platforms complicates standardization and clinical interpretation. This study compared the diagnostic performance of four chemiluminescent immunoassays (CLIAs)—Roche, Snibe, DiaSorin, and Architect, providing a comprehensive evaluation of their diagnostic performance.

Spearman’s rank correlations across the platforms were very strong, ranging from r = 0.924 to r = 0.969, underscoring high inter-assay concordance (Fig 2). Such robust correlations suggest that these platforms are generally interchangeable in routine clinical settings, although differences in sensitivity and specificity must be considered. High inter-assay concordance minimizes the risk of significant variability when switching between platforms, a critical factor in clinical laboratories managing large volumes of vitamin D testing.

Among the platforms, DiaSorin emerged as the most balanced platform, achieving both high specificity (94–99%) and sensitivity (86–98%) across comparisons (Table 1). This balance ensures reliable classification of both vitamin D sufficiency and deficiency, making DiaSorin suitable for diverse clinical applications. Bias assessment highlighted its close alignment with other assays (Fig 3), and Spearman’s correlation confirmed strong correlation with all other assays (r > 0.9, Fig 2). This robustness and balance make DiaSorin suitable for diverse clinical scenarios, including settings requiring precise classification of both vitamin D deficiency and sufficiency. Its reliability in deficiency detection is critical for managing populations at risk of adverse health outcomes associated with vitamin D deficiency [16].

**Table 1:** Summary of the diagnostic performance of the four CLIA.

TestxReference	Sensitivity	Specificity	PPV %	NPV %	ORA %	κ
**RochexSnibe**	99 (95-100)	85 (79-90)	84 (78-89)	99 (96-100)	91 (88-94)	0.82 (0.76-0.88)
**RochexDiasorin**	98 (95-100)	89 (83-93)	89 (83-93)	98 (95-100)	93 (90-96)	0.86 (0.81-0.92)
**RochexArchitect**	93 (88-96)	96 (92-99)	97 (93-99)	92 (87-96)	94 (91-97)	0.89 (0.84-0.94)
**SnibexRoche**	84 (78-89)	99 (96-100)	99 (95-100)	85 (79-90)	91 (88-94)	0.82 (0.76-0.88)
**SnibexDiasorin**	93 (88-96)	98 (95-100)	98 (95-100)	94 (89-97)	96 (93-98)	0.91 (0.87-0.96)
**SnibexArchitect**	81 (75-86)	99 (95-100)	99 (95-100)	81 (74-86)	89 (85-92)	0.78 (0.71-0.85)
**DiasorinxRoche**	89 (83-93)	98 (95-100)	98 (95-100)	89 (83-93)	93 (90-96)	0.86 (0.81-0.92)
**DiasorinxSnibe**	98 (95-100)	94 (89-97)	93 (88-96)	98 (95-100)	96 (93-98)	0.91 (0.87-0.96)
**DiasorinxArchitect**	86 (80-90)	99 (95-100)	99 (96-100)	85 (79-90)	92 (88-94)	0.83 (0.77-0.89)
**ArchitectxRoche**	97 (93-99)	92 (87-96)	93 (88-96)	96 (92-99)	94 (91-97)	0.89 (0.84-0.94)
**ArchitectxSnibe**	99 (95-100)	81 (74-86)	81 (75-86)	99 (95-100)	89 (85-92)	0.78 (0.71-0.85)
**ArchitectxDiasorin**	99 (96-100)	85 (79-90)	86 (80-90)	99 (95-100)	92 (88-94)	0.83 (0.77-0.89)

Among the platforms, Snibe demonstrated exceptional specificity (98–99%, Table 1), making it a reliable option for confirming vitamin D sufficiency. High specificity is critical in avoiding false positives, which can lead to unnecessary interventions such as supplementation, dietary restrictions, or frequent retesting. These findings are particularly relevant in the context of resource-limited settings, where overutilization of healthcare resources may reduce access for populations with genuine clinical needs.

Additionally, Snibe exhibited a median difference of 2.80 ng/mL compared to DiaSorin in the bias assessment (Fig 3), quantifying the measurement bias between the two assays. Its high correlation coefficient (κ = 0.91, Table 1) underscores Snibe’s potential as a cost-effective alternative to the more established DiaSorin platform [17,18]. This alignment also addresses the long-standing issue of variability in vitamin D measurements between assays, which has often complicated clinical interpretation and standardization efforts [19,20]. Additionally, Snibe’s operational efficiency and low reagent costs make it a cost-effective choice, particularly in large-scale health initiatives or routine population screening programs where affordability and accessibility are paramount. Despite these strengths, Snibe exhibited some variability in sensitivity (81–93%, Table 1), necessitating cautious use in identifying deficiencies, particularly in at-risk populations. Nevertheless, its specificity and cost-effectiveness make it particularly suited for follow-up vitamin D screening programs aimed at confirming sufficiency rather than initial deficiency detection.

Architect excelled in sensitivity, achieving values between 97% and 99% across all comparisons (Table 1). This makes it particularly effective for detecting true deficiencies, a critical requirement in populations at risk for severe outcomes, such as those with chronic illnesses or pregnancy complications. However, its specificity was slightly lower (81–92%, Table 1), potentially leading to false-positive sufficiency classifications. Architect’s strongest correlation with Roche (r = 0.969, Fig 2), along with a close median difference of 2.00 ng/mL in the bias assessment (Fig 3), further reinforces its reliability. These findings suggest that Architect is most suitable for deficiency screening in clinical settings where sensitivity is prioritized over specificity.

Roche demonstrated consistent performance with sensitivity ranging from 93% to 99% and specificity from 85% to 96% (Table 1). Its balanced diagnostic metrics make it a dependable choice for routine clinical testing. Bias assessment indicated a close agreement with Architect, with a median difference (Architect – Roche) of 2.00 ng/mL, as shown in Fig 3, while strong correlations with Snibe and DiaSorin (r = 0.936 and r = 0.924, respectively, Fig 2) further underscore its robustness. Roche’s reliable performance across a range of vitamin D levels ensures its utility in general diagnostic applications.

The findings of this study hold significant clinical implications. Snibe’s high specificity makes it ideal for large-scale screening programs aimed at confirming sufficiency, particularly in resource-limited settings where cost is a critical consideration. DiaSorin’s balanced sensitivity and specificity ensure accurate classification of both deficiency and sufficiency, making it suitable for diverse clinical applications, including monitoring at-risk populations and research studies. Architect’s superior sensitivity highlights its utility in identifying deficiencies in high-risk groups, while Roche provides a well-balanced option for routine diagnostic use.

In regions with high vitamin D deficiency prevalence, such as the Middle East, platforms with higher sensitivity, like Architect and DiaSorin, may be more suitable for detecting true deficiencies and preventing underdiagnosis. In contrast, highly specific platforms like Snibe can be valuable for confirming sufficiency, particularly in follow-up screenings. Optimizing assay selection based on sensitivity or specificity priorities can enhance patient outcomes and resource allocation in diverse healthcare settings.

This study has several limitations. The geographic focus may limit the generalizability of the findings to other populations. Additionally, while the study included a broad range of vitamin D levels, it did not fully account for factors such as age, gender, or comorbidities, which may influence assay performance. Importantly, LC-MS/MS, the reference standard for vitamin D measurement, was not used in this study. As such, the true diagnostic accuracy of the evaluated CLIA platforms cannot be confirmed, and findings are limited to inter-assay comparisons. Future research should incorporate more diverse demographic samples and evaluate the clinical impact of assay variability on population-level health outcomes. Moreover, longitudinal studies are needed to assess how diagnostic platform selection affects clinical decision-making and long-term patient outcomes. Finally, due to the non-parametric distribution of our data and the lack of accessible software for Passing-Bablok regression within the revision timeframe, we relied on median differences with interquartile ranges and Spearman’s rank correlation to assess assay agreement. While these methods robustly describe bias and correlation, future studies could benefit from incorporating Passing-Bablok or Deming regression to further characterize systematic and proportional biases. Additionally, detailed analytical validation data such as limits of quantitation and precision for the assays were not generated in this study, as these platforms have well-established performance profiles in the literature. This was beyond the scope of the current work but is acknowledged as valuable context for interpreting assay performance.

## 5. Conclusion

This study evaluated four chemiluminescent immunoassays: Roche, DiaSorin, Snibe, and Architect for vitamin D assessment. Snibe’s exceptional specificity and cost-effectiveness make it a valuable tool for sufficiency classification in large-scale and resource-limited settings. DiaSorin’s balanced performance ensures reliable classification across diverse scenarios, while Architect’s superior sensitivity makes it ideal for deficiency screening in high-risk populations. Roche’s consistent performance underscores its suitability for routine clinical testing. These findings emphasize the importance of aligning assay selection with clinical objectives and underscore the need for global standardization to improve the accuracy and reliability of vitamin D diagnostics.

## Supporting information

S1 Table Comprehensive vitamin D measurement data across all assays. This table presents the full dataset of vitamin D levels measured in all study samples using various assay methods.
(XLSB)
